# Effect of Direct Viral–Bacterial Interactions on the Removal of Norovirus From Lettuce

**DOI:** 10.3389/fmicb.2021.731379

**Published:** 2021-09-07

**Authors:** Zhangkai Xu, Zishu Liu, Jiang Chen, Songyan Zou, Yan Jin, Ronghua Zhang, Yaqi Sheng, Ningbo Liao, Baolan Hu, Dongqing Cheng

**Affiliations:** ^1^School of Medical Technology and Information Engineering, Zhejiang Chinese Medical University, Hangzhou, China; ^2^College of Environmental and Resource Sciences, Zhejiang University, Hangzhou, China; ^3^Zhejiang Provincial Center for Disease Control and Prevention, Hangzhou, China

**Keywords:** norovirus, lettuce microbiome, viral persistence, viral environment migration, viral-bacterial interaction

## Abstract

Norovirus (NoV) is the main non-bacterial pathogen causing outbreaks of gastroenteritis and is considered to be the leading cause of foodborne illness. This study aims to determine whether lettuce-encapsulated bacteria can express histo-blood group antigen (HBGA)–like substances to bind to NoV and, if so, to explore its role in protecting NoV from disinfection practices. Fifteen bacterial strains (HBGA-SEBs) were isolated from the lettuce microbiome and studied as they were proved to have the ability to express HBGA-like substances through indirect ELISA detection. By using attachment assay, HBGA-SEBs showed great abilities in carrying NoVs regarding the evaluation of binding capacity, especially for the top four strains from genera *Wautersiella*, *Sphingobacterium*, and *Brachybacterium*, which could absorb more than 60% of free-flowing NoVs. Meanwhile, the direct viral–bacterial binding between HBGA-like substance-expressing bacteria (HBGA-SEB) and NoVs was observed by TEM. Subsequently, results of simulated environmental experiments showed that the binding of NoVs with HBGA-SEBs did have detrimental effects on NoV reduction, which were evident in short-time high-temperature treatment (90°C) and UV exposure. Finally, by considering the relative abundance of homologous microorganisms of HBGA-SEBs in the lettuce microbiome (ca. 36.49%) and the reduction of NoVs in the simulated environments, we suggested putting extra attention on the daily disinfection of foodborne-pathogen carriers to overcome the detrimental effects of direct viral–bacterial interactions on the reduction of NoVs.

## Introduction

Norovirus (NoV) is a contagious virus that is the leading cause of foodborne illness producing high morbidity and mortality rates (Robilotti et al., 2015). Although most NoV-infected individuals have mild symptoms like vomiting and diarrhea in short duration, NoVs are still regarded as the top contributor to non-bacterial epidemic gastroenteritis in people of all ages and more often induce severe diseases of infants, young children, the elderly, and the immunosuppressed (Hassan and Baldridge, 2019; Bhavanam et al., 2020). It has been estimated that NoVs cause approximately 699 million illnesses and 219,000 deaths per year worldwide (Campillay-Véliz et al., 2020). As the pursuit of healthy living has become the interest of the entire human community, symptoms caused by NoVs have drawn more and more attention; meanwhile, there is a growing concern on how to prevent the spread and outbreak of NoVs in human-related environments.

Histo-blood group antigens (HBGAs) are identified as the specific cellular receptors for most genotypes of human NoVs in the past decade (Lindesmith et al., 2003; Gaythorpe et al., 2018; Esseili et al., 2019), which specifically bind with the P2 subdomain of NoVs protruding domain. Also, they played a key role in the interactions of NoVs with surrounding organisms during propagation (Deng and Gibson, 2017; Amarasiri and Sano, 2019). The chemical nature of HBGAs are complex terminal carbohydrates (Sestak, 2014) and are classified into types A, B, H, and Lewis depending on the terminal glycosyl group, which differ in their ability to bind NoV particles (Almand et al., 2017a). HBGAs are not only present on human cells, i.e., red blood and mucosal epithelial cells, but also secreted into bodily fluids, i.e., blood, saliva, and intestinal secretions (Ravn and Dabelsteen, 2000; Ramani and Giri, 2019).

Apart from these, enteric bacteria isolated from the stool could express HBGA-like substances (i.e., polysaccharides) (Miura et al., 2013; Almand et al., 2017c), which also had the capacity of directly binding to NoVs, and promoted NoVs to stay and infect host cells in the gut system (Jones et al., 2014; Monedero et al., 2018). In addition, HBGA-like substances were also found in extracellular polymeric substances (EPS) and lipopolysaccharides (LPS) secreted by human intestinal bacteria harvested from *in vitro* environments, like aquatic ecosystems (Esseili et al., 2012; Le Guyader et al., 2012; Amarasiri and Sano, 2019). It means that the health risk of NoVs might be exacerbated due to the large reservoir of HBGA expressed bacteria in the environment.

Direct interactions between eukaryotic viruses, for instance, NoVs and bacteria, have recently become a hot issue, in which viral infectivity and stability can be facilitated by binding to bacterial cells or by using bacterial products (Moore and Jaykus, 2018). At the same time, there is growing evidence that such direct viral–bacterial interactions were widespread in the environment (Almand et al., 2017b). For example, Li et al. (2015) reported that when compared with a strain of *Escherichia coli* with no expression of HBGA-like substances, two expressed *E. coli* strains significantly protected the receptor-binding ability of NoVs within 2 min of heat treatment.

Extending from this point, the presence of HBGA/HBGA-like substances on food products has drawn the attention of researchers. Binding capacity was studied to examine whether the specific binding mechanisms of NoVs are existing (Amarasiri and Sano, 2019; Eshaghi Gorji et al., 2021). Esseili et al. (2012) discovered the direct attachment of NoVs to lettuce leaves (e.g., along veins and stomata) using immunofluorescence and ELISA composite approach and proved the binding was specific to cell wall carbohydrates which associated with HBGAs. In the following, Liu et al. (2020) demonstrated, with a similar approach, that the bacterial cultivation of *Pseudomonas mosselii* originating from the lettuce surface had a high ability to bind multiple NoV particles including types GI and GII by high-level expressing HBGA-like substances. As NoVs were suspected to interact with bacteria *via* the binding on HBGA-like substances, and this might be one of the main reasons for the foodborne transmission of NoVs, it deserves to explore the mechanisms behind it.

Foodborne transmission is a substantial route for the spread of NoVs in complement to the fecal–oral transmission. Especially where food products were exposed to contaminated water and air, e.g., farms and farmlands downstream of sewage treatment plant outfalls (Callejón et al., 2015; Gonzales-Gustavson et al., 2019), NoVs encounter these microbes *via* aerosol and irrigating water, and thus cause a certain impact on its subsequent dissemination in the environment or food chain. As a primary foodborne carrier responsible for outbreaks of NoVs, lettuce houses highly diverse microbes including bacteria, fungi, and protozoa that are collectively referred to as the microbiome (Moore and Jaykus, 2018; Amarasiri and Sano, 2019; Campillay-Véliz et al., 2020). To date, the interaction between NoVs and commensal bacteria is only known for a few bacterial species (Miura et al., 2013; Li et al., 2015), and most of the binding is mediated through HBGA-like substances. However, the impact of this kind of interaction on the stability of NoVs, e.g., infective persistence and resistance to the unfavorable environment, remains to be further investigated.

Based on these various studies, the hypothesis that NoVs can bind to HBGA-like substance-expressing bacteria (HBGA-SEB) and the binding can affect the stability of NoVs on lettuce came up in this study. With focuses on the viral–bacterial interaction by isolated bacteria identification and verifying their NoV-binding capacity, the effects of interspecies interactions on the disinfection efficiency of NoVs bound to lettuce are studied by simulating daily disinfection means through high-temperature rinsing and UV irradiation. This study aims to provide a new perspective for exploring the viral–bacterial relationship and to provide an experimental basis for preventing and controlling the food-borne transmission of NoVs.

## Materials and Methods

### Isolation and Cultivation of Bacteria From Lettuce

Bacteria used for taxonomy identification were isolated from romaine lettuce (i.e., *Lactuca sativa* L. var. *longifolial*), which was harvested from outdoor farmland at Hangzhou, China (30°11′39.40N, 120°10′4.87E) as described by Liu et al. (2020). Briefly, 20-g lettuce samples were cut into 1.5-cm^2^ pieces and washed in a sterile conical flask with 200 ml PBS (pH = 7.2) at 180 rpm for 20 min at room temperature. Then the washing mixture was filtered through a double-layer gauze, and the filtrate was dispensed into 5-ml aliquots. One aliquot was taken and centrifuged at 3,000 × *g* for 5 min and harvested the cell pellet for bacterial isolation. The cell pellet was resuspended with 500 μl sterilized PBS, and a dose of 50-μl cell suspension was partitioned and scribed on a 0.1× TSA plate (Hope Bio, Qingdao, China) as the inoculation of lettuce-derived bacteria. Inoculated TSA plates were cultivated at 25°C for 7 days, during which time single colonies were picked for the following isolation and purification. The purified isolates were harvested after several transfers and stored in skimmed milk at −80°C.

### Detection of HBGA-Like Substance Expression

Purified isolates were inoculated in 0.1 × TSB liquid medium (Hope Bio) as a single colony per flask and cultivated at 25°C at 120 rpm for 18 h to detect the expression of HBGA-like substances. Bacterial cultivation (4 ml) was taken and centrifuged at 3,000 × *g* for 2 min. After removing the supernatant, the cell pellet was resuspended in sterilized PBS, vortexed at 2,000 rpm for 20 min, and then centrifuged at 10,000 × *g* for 2 min. The supernatant was collected to detect the expression of HBGA-like substances.

Expressed HBGA-like substances per isolation were quantified based on the indirect ELISA approach that was modified from a previously published protocol (Miura et al., 2013; Almand et al., 2017c). In brief, the ELISA plate was incubated with 200 μl of the stored supernatant per well at 4°C overnight. PBS (200 μl) was used as the negative control (NC) and triplicate wells were prepared for each sample and the NC. Wells were washed with PBS three times and blocked with 5% bovine serum albumin (BSA) at 37°C for 1 h. Afterward, wells were washed three times with PBS. Then 100 μl of HBGA monoclonal antibodies (MAbs, diluted to 1:1,000 with PBS containing 5% BSA; Covance, United States) was added to all wells and incubated at 37°C for 1 h. HRP-conjugated goat anti-mouse IgG (H+L chains, diluted to 1:10,000 with PBS containing 5% BSA; Yeasen, Shanghai, China) and HRP-conjugated goat anti-mouse IgM (H+L chains, diluted to 1:10,000 with PBS containing 5% BSA; Yeasen) were used as the secondary antibodies. The wells were washed three times with PBST after each antibody incubation. One hundred microliters of TMB (Sangon Biotech, Shanghai, China) was added to each well. After incubating in the dark for 5–25 min, the chromogenic reaction was halted using 50.0 μl of 2.0 mol/L H_2_SO_4_. The absorbance per well at 450 nm was measured by Spark 10M multimode microplate reader (TECAN, Switzerland), and if the ratio of mean absorbance (S/N) of a sample (S) to the negative control (N) was greater than 2, the bacteria corresponding to this sample were defined as HBGA-SEB.

Results of indirect ELISA for each isolated strain are shown in Supplementary Table 1; according to the S/N values, 15 HBGA-SEB strains were determined. Also, HBGA-SEB strains and the bacterial composition of the lettuce-derived microbiome were identified by the 16S rRNA gene amplicon sequencing. Detailed steps for DNA extraction and PCR reaction are given in Supplementary Material 2. Also, primers used for the community composition analysis and HBGA-SEB identification are listed in Supplementary Tables 2, 3, respectively.

### Determining the Binding Capacity by the Viral–Bacterial Attachment Assay

Single colony for each bacterial strain expressing HBGA-like substances was picked and cultivated overnight with 0.1 × TSB liquid medium at 120 rpm and 25°C. After cultivation, 1 ml of bacterial culture was centrifuged at 10,000 × *g* for 30 s to collect the cell pellet, and then the cell pellet was washed with sterilized PBS. The washing steps were repeated twice and the OD (600 nm, λ = 1 cm) of the cell suspension was adjusted to 1.0 with sterilized PBS. OD-adjusted solution was used as the bacterial suspension when mixed with the NoV suspension to test the binding capacity.

The suspension of virus particles was made from the stool of a NoV GII.4–positive clinical gastroenteritis patient provided by the Zhejiang Provincial Center for Disease Control and Prevention. The stool was first mixed with an appropriate amount of sterilized PBS to obtain a 10% fecal mixture. After being vortexed well until homogeneous, the fecal mixture was centrifuged at 3,000 × *g* for 10 min, carefully pipetting out the supernatant and used as the NoV suspension.

To test of binding capacity, 100 μl bacterial suspension for each HBGA-SEB was mixed with 100 μl NoV suspension identically; *E. coli* ATCC15597 suspension was used as a bacteria control (EC) as it had no expression of HBGA-like substance (Miura et al., 2013). The volume of the bacterial–viral mixture was fixed to 500 μl with sterilized PBS and incubated at room temperature for 2 h. After the bacterial–viral mixture was centrifuged at 10,000 × *g* for 5 min, 100 μl supernatant was pipette out for viral nucleic acid extraction with QIAamp Viral RNA Mini Kit (QIAGEN, CA, Germany) by following the instruction manual. The extracted RNA was used as the template for reverse transcription-quantitative real-time PCR (RT-qPCR) detection with One Step Primescript RT-PCR Kit (Takara, CA, Japan). The information of primers and probes, as well as the RT-qPCR reaction procedure are shown in Supplementary Material 3.

RT-qPCR reaction was performed on the ABI 7500 Fast Real-Time PCR system (ABI, United States) and the CT value was converted into the viral copies according to an established method (Cheng et al., 2018). The binding capacity η for each HBGA-SEB was quantified as described in Eq. 1:

(1) $$\eta \left(\%\right)=100\%\times \left(C_{0}-C_{X}\right)/C_{0}$$

Where: *C*_0_ is the viral copies of the original NoVs suspension;

*C*_*X*_ is the viral copies of the supernatant after HBGA-SEB absorption.

Bacteria with the biggest η value were checked with transmission electron microscopy (TEM) to visualize the direct binding between bacteria and NoV particles, for which 100 μl bacterial suspension was centrifuged at 3,000 × *g* for 5 min, and the cell pellet was collected and resuspended in 100 μl NoV suspension. Then the viral–bacterial mixture was incubated at room temperature for 2 h, before checking with TEM (H-7650; HITACHI, Japan).

### Reduction of NoVs Under the Simulated Environments

The viral–bacterial mixture was made by mixing 50 μl HBGA-SEB suspension with 50 μl NoV suspension in a 200-μl EP tube and then incubated at room temperature for 2 h. In addition, sterilized PBS was used as a NC and *E. coli* ATCC15597 suspension was used as a EC for the reduction analysis. A series of treatments were made based on the viral–bacterial mixture to test the effect of simulated environmental conditions on the reduction of NoVs, which was determined by calculating the log reduction as Eq. 2. As the equation indicates, the higher the –log reduction (*N*_*t*_/*N*_0_) value, the higher the NoV reduction (Fuentes et al., 2020). NoVs were quantified before and after the treatment by the RT-qPCR approach.

(2) $$V\,i\,r\,u\,s\,r\,e\,d\,u\,c\,t\,i\,o\,n=-L\,o\,g\,r\,e\,d\,u\,c\,t\,i\,o\,n\,\left(\frac{N_{t}}{N_{0}}\right)$$

Where: *N*_0_ is the viral copies of NoVs before treatment;

*N*_*t*_ is the viral copies of NoVs per sample after treatment.

Norovirus reduction was calculated after the treatments for both the viral–bacterial mixtures and the virus–bacteria–lettuce combinations. The viral–bacterial mixture was treated under two different simulated environments to investigate the effects of daily disinfection methods on the NoV reduction: (1) hot rinsing, i.e., 90°C (metal bath; ABI 7500 Fast Real-Time PCR system, ABI); (2) UV irradiation, i.e., 0.11 mW/cm^2^ (Philips 30 W Ultraviolet-C lamp, irradiating with a 65-cm distance to the sample).

For the virus–bacteria–lettuce combination, first, the surface of lettuce was rinsed with distilled water and displayed in the biosafety cabinet to dry. Then the surface of the lettuce leaves was irradiated with UV light for 10 min. Afterward, small areas were marked on the surface of lettuce leaves in parallel for spreading evenly the viral–bacterial mixture. Finally, the virus–bacteria–lettuce combination was treated under two different simulated environments: (1) switch incubation at 37 and 25°C for 12 h each for 7 days (SHP250; SENXIN, China) to investigate the reduction of NoVs on the lettuce during normal storage; (2) UV irradiation to investigate the effect of UV disinfection on the reduction of NoVs from lettuce. After the treatment, the marked areas of leaves were cut into pieces and put into a 1.5-ml sterile EP tube with 1 ml RNase-free water. Then the EP tube was shaken 10 times per second for 10 min in a pre-cooled (4°C) ball mill before centrifugation at 500 × *g* for 60 s, and the supernatant was taken for the viral nucleic acid extraction.

### Statistical Analysis

Triplicate measurements are performed as the biological replicates for each experiment. Numeric variables were presented as the mean ± SD, and the significance of the variation between groups was examined by *t*-test, while *p*-value <0.05 was considered as a statistically significant variation between groups. All the data were analyzed by the software SPSS 19.0.

## Results

### The Binding Capacity of HBGA-SEB to NoVs

**FIGURE 1:**
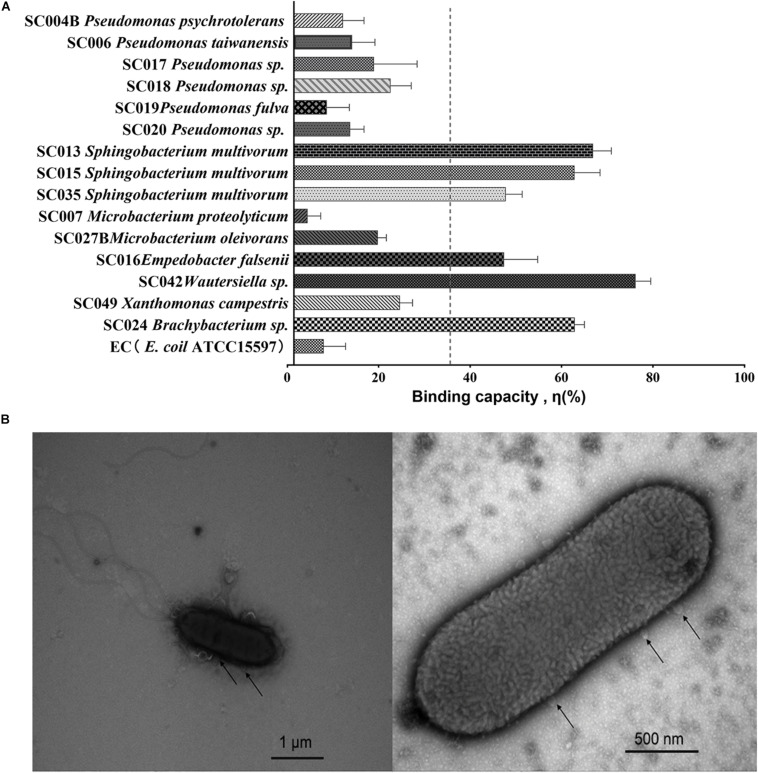
Viral–bacterial binding between noroviruses (NoVs) and HBGA-SEB. **(A)** Binding capacity η (%) of HBGA-SEB strains to NoVs; the EC is the bacterial control of *Escherichia coli* ATCC15597, which has no HBGA-like substance expression; the average binding capacity η_mean_ = 34.22% is marked as the vertical dash line. **(B)** Transmission electron microscopy (TEM) images indicate the direct binding between SC042 cells and NoV (GII.4) particles.

The binding capacity η was analyzed for each of the 15 HBGA-SEBs and the EC *E. coil* ATCC15597 (EC, Figure 1A) through a viral–bacterial attachment assay. Compared with the EC, which had no expression of HBGA-like substances, most of the HBGA-SEB (14 in 15 strains) showed extra binding capacities. Regarding the average binding capacity of all HBGA-SEB except SC007, η_mean_ = 34.22%, six HBGA-SEB strains had relatively high binding capacity, among which SC042 showed the highest binding capacity with η = 74.81 ± 3.29%; the others in descending order were SC013 (65.48 ± 4.00%), SC024 (61.49 ± 2.08%), SC015 (61.43 ± 5.56%), SC035 (46.39 ± 3.56%), and SC016 (46.03 ± 7.32%).

Previously published TEM images have shown human NoVs could bind to multiple areas around the commensal bacteria such as the extracellular polysaccharide matrix (EPS), the outer membrane, pili, and flagella (Miura et al., 2013; Almand et al., 2017c; Madrigal et al., 2020). With a similar pipeline, TEM is used to visualize the direct binding between NoV particles and the HBGA-SEB, where the attachment assay of NoVs (GII.4 suspension) and SC042 has been chosen with regard to the highest binding capacity (Figure 1B).

### Identification of HBGA-SEB Originated From Lettuce Microbiome

**FIGURE 2:**
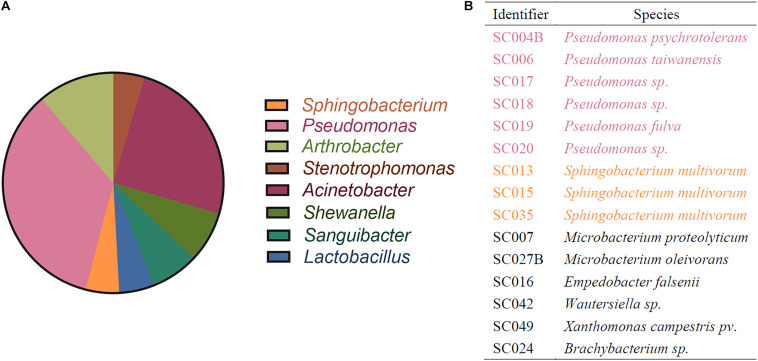
Identification of bacteria by 16s rRNA sequencing. **(A)** Community composition of lettuce microbiome resolved in genus level; only genera with relative abundance >1% are shown. **(B)** Identification of HBGA-SEB strains.

To isolate HBGA-SEB strains from the lettuce microbiome, a native microbial community was harvested from the randomly picked lettuce leaves, and the community composition is shown at the genera level in Figure 2A. Based on this native microbial community, 15 bacterial isolations that had high abilities to express HBGA-like substances (determined by the indirect ELISA approach; see Supplementary Material 1) were identified, and their taxonomy is shown in Figure 2B, among which six *Pseudomonas* spp. and three *Sphingobacterium* spp. cover more than 60% of all HBGA-SEB strains, and the genus they belong to occupied a fraction of 39.64% of all abundant genus harvested from the native lettuce microbiome (i.e., relative abundance of genus >1%; Figure 2A). The detection of *Pseudomonas* spp., which was well known for its ability in expressing HBAG-like substances, was consistent with the results of previous studies (Rastogi et al., 2012; Erlacher et al., 2014; Deng and Gibson, 2017; Liu et al., 2020). This indicated a certain number of bacteria on the surface of lettuce might have high abilities to express HBAG-like substances. However, their effects on NoV reduction need to be further studied.

### Effects of Viral–Bacterial Interaction on NoV Reduction

To investigate the influence of viral–bacterial interaction on NoV reduction, HBGA-SEB strains from three different genera were selected to be further studied: SC006, SC015, and SC042. SC006 was a *Pseudomonas* sp. that had five types of HBGA-like substances highly expressed with the highest expressed Lewis B type of all HBGA-SEBs (Supplementary Table 1); SC015 was a *Sphingobacterium* sp. which also had five types of HBGA-like substances highly expressed, and with the highest expression of B type; SC042 was a *Wautersiella* sp. which had four types of HBGA-like substances highly expressed, with the highest expression of both types A and H. Although the genera of SC042 were uncommonly found in the native lettuce microbiome, it was still selected as it had the highest binding capacity (Figure 1A). In addition to these HBGA-SEBs, sterilized PBS buffer was used as a NC, and the *E. coli* ATCC15597 was used as a EC, as it was known that it had no expression of HBGA-like substance, in testing the NoV reduction under simulated scenarios.

**FIGURE 3:**
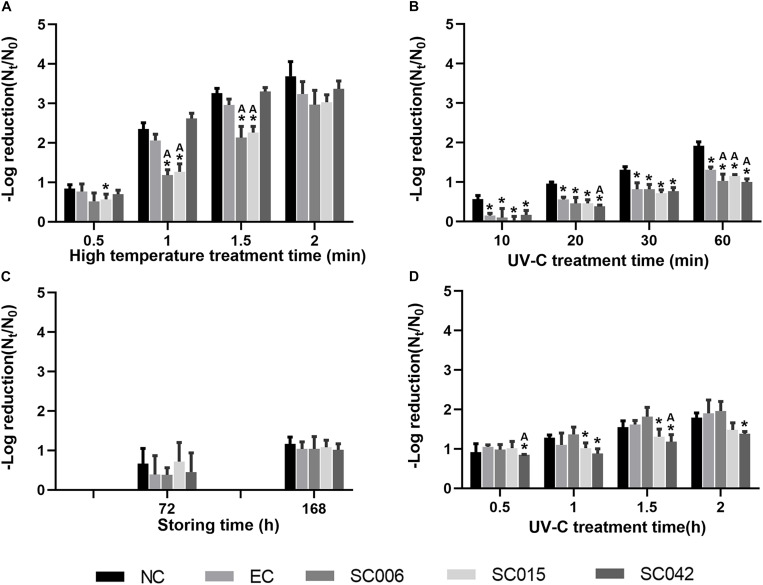
Effects of viral–bacterial interaction on NoV reduction. **(A)** Simulation of hot rinsing disinfection; **(B)** simulation of UV irradiation disinfection; **(C)** adherence on the lettuce and simulation of normal storage; **(D)** adherence on the lettuce and simulation of UV irradiation. NC means negative control, which is PBS buffer without bacterial cells; EC means bacterial control, which is prepared with *E. coli* ATCC15597 suspension. The * symbol means significantly varied from the NC group. The “A” symbol means significantly varied from the EC group. Students’ *t*-test is used for the significance testing, *p* < 0.05.

At first, viral–bacterial mixtures were treated alone under simulated environments, i.e., hot rinsing (90°C) and UV irradiation, to investigate the effects of general disinfection methods on NoV reduction, and results are shown in Figures 3A,B, respectively. The value of –log reduction (*N*_*t*_/*N*_0_) represents the reduction of virus caused by the treatment, and the higher the value, the greater the reduction. The result showed that hot rinsing was significantly more efficient in removing NoVs than UV irradiation, and the reduction caused by 1 min 90°C hot rinsing (Figure 3A) was greater than 60 min UV-C irradiation (Figure 3B). In addition, the interaction between NoVs and selected HBGA-SEBs had caused negative impacts on NoV removal. For instance, in the situation of 1–1.5 min of hot rinsing, the reduction of NoVs attached to SC006 and SC015 was significantly (*t*-test, *p* < 0.05) less than they were in NC, EC, and SC042 (Figure 3A). Also, the reduction of NoVs attached to SC006, SC015, and SC042 were significantly less than they were in NC and EC when treated under UV irradiation for 60 min (Figure 3B).

Furthermore, the effects of adhering viral–bacterial mixtures to the surface of lettuce on the reduction of NoVs were investigated. For the virus–bacteria–lettuce combinations, two simulated environments were built to test the effects of viral–bacterial interaction on NoV reduction. One treatment was to simulate summer storage conditions by switching the temperature between 37 and 25°C every 12 h, as with diurnal temperature changes. Low reduction of NoVs was found even after 168 h of incubation under this condition [*−log_10_*(*N*_*t*_/*N*_0_) = 1.07, Figure 3C], while the influence of viral–bacterial interaction on the NoV reduction was not significant. The other treatment was to simulate the UV disinfection process for vegetables. Higher reductions were found for UV treatment than the alternating temperature [average *−log_10_*(*N*_*t*_/*N*_0_) = 1.13, 1.50, and 1.70 at 1, 1.5, and 2.0 h, respectively; Figure 3D]. After 1 h of UV irradiation, the reduction of NoVs in SC015 and SC042 was less than they were in NC and EC, but such differences were only significant in SC042 with 1.5 h of UV irradiation.

## Discussion

### HBGA-SEB Contribute to the High Affinity Between NoVs and Lettuce

Noroviruses are major causative agents of non-bacterial acute gastroenteritis, which constitute a substantial disease burden worldwide (De Graaf et al., 2016; Mackowiak et al., 2018). HBGAs/HBGA-like substances have been recognized as the critical receptors/co-receptors for NoVs (Huang et al., 2005; Almand et al., 2017a). In this study, 15 lettuce-microbiome-isolated bacteria have been determined as HBGA-SEBs regarding their high abilities to express HBGA-like substances. The result of attachment assay testing revealed that HBGA-SEBs have generally higher binding capacities compared with the non-HBGA-expressing EC *E. coli* ATCC15597 (except the SC007 *Microbacterium proteolyticum*), and 4 of these 15 HBGA-SEBs even have binding capacities greater than 60%.

Comparing the taxonomic identity of HBGA-SEB with the member of the native lettuce microbiome (Figure 2), homologous bacteria of HBGA-SEBs (i.e., six *Pseudomonas* spp. and three *Sphingobacterium* spp.) almost occupied 40% of all dominant genera in relative abundance. *Pseudomonas* is the genus with the most isolated strains and the most abundant genus in the initial lettuce microbiome (Figure 2). This result was consistent with previous studies as *Pseudomonas* spp. were reported to be dominant in the leafy microbiome of lettuces (Rastogi et al., 2012; Erlacher et al., 2014). Our finding could suggest the high natural affinity between NoVs and lettuce, as members of the lettuce microbiome could express HBGA-like substances and acted like vehicles for the environmental spreading of NoVs (Liu et al., 2020).

### Detrimental Effects of Viral–Bacterial Interaction on the Reduction of NoVs

Noroviruses are highly resistant to environmental disturbances, e.g., heat, chlorine disinfection, and UV radiation (DiCaprio et al., 2015), and the persistence of infectivity can be further promoted by the binding of NoVs on surfaces of other objects and microorganisms. In the previous simulation study, the detrimental effects on NoV reduction have been observed with HBGA-like substance expressed *E. coli* and recombinant baculoviruses containing the VP1 protein from NoVs GI.1 and GII.4 (Li et al., 2015).

In this study, mixed suspensions of NoV particles and HBGA-SEBs (SC006, SC015, and SC042, respectively) were treated similarly, as Li et al. (2015) did to simulate the daily disinfection, i.e., hot water rinsing and UV irradiation. Also, the virus–bacteria–lettuce combinations were treated under the simulated summer storing condition and UV disinfection. Our results confirmed that direct viral–bacterial interactions have significant detrimental effects on short-time treatments, regardless of disinfected methods, and a limited reduction of NoVs for virus–bacteria–lettuce combinations with lettuce-isolated bacteria and real human NoV particles. In such conditions, bacteria-expressing HBGA-like substances while binding to NoVs (Zhang et al., 2021) formed a barrier, thus protecting NoVs from the influence of external disturbances. While SC006 (*Pseudomonas taiwanensis*) and SC015 (*Sphingobacterium multivorum*) were more effective in assisting NoVs to resist heat (Figure 3A), SC042 (*Wautersiella* sp.) was more effective to resist UV irradiation (Figure 3B); this might be related to the levels to which the strains express different HBGAs (Supplementary Material 1 and Supplementary Table 1).

In addition to this, we found that under the same processing conditions (i.e., 30 and 60 min of UV irradiation, Figures 3B,D) the NoV reduction in virus–bacteria–lettuce combination was lower than that in virus–bacteria mixtures. This may be due to the possible production of HBGA-like substances by lettuce leaf that could also bind to and protect NoVs (Esseili et al., 2019). Li et al. (2011) have reported a similar result; while facing UV irradiation, the presence of lettuce can reduce the inactivation of viruses. However, it cannot be denied that this may be caused by a more complex coupling mechanism between virus, bacteria, and lettuce, which needs to be further investigated.

### Daily Disinfections Might Be Insufficient to Prevent NoVs

Interhuman transmission is the main pathway of NoV spreading, as the CDC has confirmed that more than 62% of NoV outbreaks are transmitted through person-to-person contact (Wikswo and Hall, 2012). NoVs can infect hosts with few viral particles (<20) (Hassan and Baldridge, 2019) and have high resistance to the harsh environment. Studies show that NoVs could remain infectious at pH 2.7, 60°C, and free chlorine concentration of 3.75–6.25 mg/L for days, and could keep the coat protein intact for 2 weeks on the surface of public facilities and 2 months in water (Seitz et al., 2011), and thus have high risks to cause secondary dissemination (Atmar et al., 2008; Teunis et al., 2008). To protect public safety, the maximum additional burden related to water and wastewater was regulated to provide an adequate margin to waterborne diarrheal diseases like NoVs (Mara, 2011). Except for water, recent studies have reported a non-negligible role of the natural microbiome in the foodborne NoV outbreaks (Marionneau et al., 2002; Gao et al., 2016). NoVs on lettuce surface are associated with irrigation water and have a certain risk of infecting people (Gonzales-Gustavson et al., 2019). Due to this, the daily disinfection for contacted water bodies and raw eaten vegetables needs special attention, as they might be key nodes in blocking the spread of NoVs.

According to a previous study, NoVs were detected in agricultural irrigation water and lettuce surface at 530 virus particles/L and 630 virus particles/g, respectively (El-Senousy et al., 2013); roughly estimated targets for the reduction of NoVs (<20 virus particles) in irrigation water and on lettuce can be derived, which are –log(*N*_0_/*N*_*t*_) = 1.42 and 1.49, respectively. In comparison with the observed reduction of NoVs in simulated environments (Figure 3), it could be seen that only hot water rinsing has a sufficient efficiency for the reduction of NoVs with HBGA-SEB mixing, but still needs more than 90 s. In the case of lettuce leaves, moderate-temperature storage for 7 days still could not meet the expected target (Hirneisen and Kniel, 2013). Meanwhile, although it is met by UV irradiation for more than 1.5 h, this treatment condition would be difficult to be implemented in the actual product due to the cost. Therefore, extra attention should be paid to the daily disinfection of food, especially for the vegetables that are usually eaten raw. Also, it is important to develop better disinfection methods for fresh produce.

To conclude, 15 HBGA-SEB strains were isolated from the lettuce microbiome and their high abilities in expressing HBGA-like substances were proved by indirect ELISA. Through attachment assay and RT-PCR approach, the binding capacity is determined for each HBGA-SEB, while in comparison with the non-HBGA-expressing *E. coli*, generally higher binding capacities were found for HBGA-SEBs. In addition, the specific attachment is directly observed between NoVs and SC042 as the support to the highest binding capacity. Subsequently, through simulated environmental experiments, it is found that with the detrimental effect of viral–bacterial interactions, routine disinfection strategies are limited in their ability to remove NoVs, especially when lettuce exists and plays a role in the interaction. To further verify the findings of this study and gain insight into the molecular mechanisms of such viral–bacterial binding, culturable NoV surrogates such as MNV-1 and TV should be examined in the future.

## Data Availability Statement

The raw data supporting the conclusions of this article will be made available by the authors, without undue reservation.

## Author Contributions

ZX, ZL, YS, BH, and DC: conceptualization. ZX, JC, SZ, YJ, RZ, and DC: methodology. ZX and ZL: writing—original draft preparation. BH, NL, and DC: review and editing. YS and DC: project administration and funding acquisition. All authors have read and agreed to the published version of the article.

## Conflict of Interest

The authors declare that the research was conducted in the absence of any commercial or financial relationships that could be construed as a potential conflict of interest.

## Publisher’s Note

All claims expressed in this article are solely those of the authors and do not necessarily represent those of their affiliated organizations, or those of the publisher, the editors and the reviewers. Any product that may be evaluated in this article, or claim that may be made by its manufacturer, is not guaranteed or endorsed by the publisher.

## Supplementary Material

Supplementary Material 1 Determining HBGA-SEB by indirect ELISA.

Supplementary Material 2 Bacterial identification by 16S rRNA sequencing.

Supplementary Material 3 Quantification of NoVs by RT-PCR.

Supplementary Material 4 Supplementary Table 7, amplicon sequences used for HBGA-SEB identification.

Supplementary Material 5 Lettuce leaf surface microbiome.fastq.
