# Supplementary material for: Effect of Direct Viral–Bacterial Interactions on the Removal of Norovirus From Lettuce
Source: Front Microbiol. 2021 Sep 7;12:731379. doi: 10.3389/fmicb.2021.731379 (PMC8453150; doi:10.3389/fmicb.2021.731379)
Supplement: Supplementary Material 1 — Determining HBGA-SEB by indirect ELISA. [file Data_Sheet_1.DOCX]

Supplementary Materials

**Effect of Direct Virus-Bacteria Interactions on the Removal of Norovirus from Lettuce**

Zhangkai Xu^1†^, Zishu Liu^2†^, Jiang Chen^1^, Songyan Zou^1^, Yan Jin^1^, Ronghua Zhang^3^, Yaqi Sheng^2^, Ningbo Liao^3^*, Baolan Hu^2^, Dongqing Cheng^1^*

1 School of Medical Technology and Information Engineering, Zhejiang Chinese Medical University, Hangzhou 310053, China; xchxzk@163.com (Z.X.); jchen@zcmu.edu.cn (J.C.); 15990075450@126.com (S.Z.); 770617423@qq.com (Y.J.);

2 College of Environmental and Resource Sciences, Zhejiang University, Hangzhou 310058, China; liuzishu@zju.edu.cn (Z.L.); blhu@zju.edu.cn (B.H.); shengyaqi@zju.edu.cn (Y.S.);

3 Zhejiang Provincial Center for Disease Control and Prevention, Hangzhou 310052, China; rhzhang@cdc.zj.cn (R.Z.); liaoningbo2010@126.com (N.L.);

† These authors contributed equally

* Correspondence: liaoningbo2010@126.com (N.L.); chengdq@zcmu.edu.cn (D.C.)；

**Contents**

[S1: Determine HBGA-SEB by indirect ELISA 2](#_Toc78556033)

[S2: Bacterial identification by 16S rRNA sequencing 4](#_Toc78556034)

[S3: Quantification of NoVs by RT-PCR 5](#_Toc78556035)

S1: Determine HBGA-SEB by indirect ELISA

Take 4 mL of bacterial suspension and centrifuge at 3000 × g for 2 min. Remove the supernatant and the resuspend the cell pellet in sterilized PBS. Vertex the cell suspension at 2000 rpm for 20, and it is followed by a centrifugation step at 10000 × g for 2 min, take the supernatant (containing bacterial EPS) for the HBGA detection.

HBGAs are detected with an indirect ELISA approach as follow: add 200 μL of the stored supernatant per each well on the ELISA plate and incubate at 4℃ overnight, in addition, 200 μL PBS is used as the negative control. Triplicate wells are prepared per each sample and the negative control. Each well was washed with PBS for three times and blocked with 5% bovine serum albumin (BSA) at 37℃ for 1 h, afterwards wells are washed once three times with PBS. 100 μL HBGAs monoclonal antibodies (MAbs, diluted to 1:1000 with PBS containing 5% BSA; Covance, USA) were added to all wells. After incubation at 37℃ for 1h, wash wells three times with PBST. HRP-conjugated goat anti-mouse IgG (H+L chains, diluted to 1:10000 with PBS containing 5% BSA; Yeasen, Shanghai, China) and HRP-conjugated goat anti-mouse IgM (H+L chains, diluted to 1:10000 with PBS containing 5% BSA; Yeasen, Shanghai, China) are used as the secondary antibodies. Measure the absorbance per well and if the If the ratio of mean absorbance (S/N) of a sample (S) to a negative control (N) is greater than 2, the bacteria corresponding to this sample are defined as HBGA-SEB. Results of indirect ELISA can be seen in Table S1, while 15 HBGA-SEB strains are determined accordingly.

**Table S1:** Results of indirect ELISA (S/N) per each isolated bacterium from the lettuce microbiome. For each strain, expressed HBGAs (S/N > 2) are filled with gray, and if the expression is the highest of all strains it is filled with blue.

|  | Precursor | A | B | H | Lewis A | Lewis B | Lewis X | Lewis Y |
| --- | --- | --- | --- | --- | --- | --- | --- | --- |
| SC003 | 1.87 | 1.33 | 1.47 | 1.05 | 0.76 | 1.41 | 0.85 | 0.76 |
| **SC004B** | 2.27 | 2.42 | 1.64 | 1.8 | 1.73 | 1.26 | 1.17 | 1.27 |
| **SC006** | 0.65 | 1.56 | 3.37 | 2.85 | 4.13 | 3.63 | 2.25 | 1.23 |
| **SC007** | 0.83 | 2.45 | 3.07 | 1.71 | 1.61 | 2.89 | 1.13 | 1.14 |
| SC009 | 1.55 | 0.99 | 1.77 | 1.9 | 1.71 | 1.42 | 0.91 | 0.54 |
| **SC013** | 0.94 | 1.26 | 1.33 | 1.91 | 1.22 | 2.05 | 3.07 | 2.12 |
| **SC015** | 1.55 | 2.99 | 4.44 | 2.17 | 2.5 | 3.35 | 0.79 | 1.08 |
| **SC016** | 1.31 | 1.39 | 2.33 | 1.91 | 2.43 | 2.07 | 2.09 | 1.07 |
| **SC017** | 1.11 | 2.29 | 1.33 | 2.41 | 1.31 | 1.03 | 2.39 | 1.04 |
| **SC018** | 3.43 | 1.61 | 1.86 | 2.96 | 1.53 | 1.49 | 1.33 | 2.92 |
| **SC019** | 1.37 | 1.73 | 1.99 | 1.39 | 1.67 | 2.26 | 1.18 | 1.17 |
| **SC020** | 1.08 | 1.29 | 2.05 | 2.67 | 1.69 | 1.22 | 0.71 | 0.62 |
| SC021 | 0.92 | 1.27 | 0.81 | 1.18 | 0.8 | 0.94 | 0.69 | 0.51 |
| SC023 | 1.22 | 1.11 | 1.19 | 1.03 | 1.02 | 1.05 | 0.9 | 1.33 |
| **SC024** | 0.68 | 3.19 | 2.26 | 2.81 | 5.09 | 2.69 | 1.75 | 1.35 |
| SC025 | 1.21 | 1 | 0.71 | 0.91 | 0.71 | 0.68 | 0.55 | 0.47 |
| SC027A | 1.24 | 1.73 | 0.87 | 1.41 | 0.67 | 0.63 | 0.61 | 0.54 |
| **SC027B** | 1.1 | 1.78 | 1.7 | 1.99 | 2.46 | 1.87 | 1.34 | 0.79 |
| SC028 | 1.19 | 1.32 | 0.99 | 0.87 | 0.74 | 0.71 | 0.69 | 0.62 |
| **SC035** | 0.89 | 1.65 | 2.09 | 2 | 1.35 | 0.65 | 0.59 | 0.52 |
| SC037 | 1.17 | 1.39 | 1.6 | 1.49 | 0.97 | 0.65 | 0.61 | 0.53 |
| SC040 | 0.84 | 1.68 | 1.38 | 1.71 | 1.01 | 0.55 | 0.5 | 0.41 |
| **SC042** | 1.21 | 4.66 | 3.19 | 4.11 | 4.33 | 1.58 | 1.1 | 1.36 |
| SC048 | 1.15 | 1.75 | 1.4 | 1.84 | 1.61 | 1.43 | 1 | 0.46 |
| **SC049** | 3.13 | 3.05 | 1.28 | 2.4 | 3.13 | 2.03 | 1.14 | 1.63 |

S2: Bacterial identification by 16S rRNA sequencing

The DNA of bacteria was extracted with AxyPrep Multisource Genomic Miniprep DNA Kit (CORNING, USA) according to the manual book. The PCR cycling profile used was 94 ℃ for 5 min, 94 ℃ for 10 s, 54 ℃ for 10 s by 35 cycles, and 72 ℃ for 7 min.

**TableS2** Primer sequence for 16S rRNA identification of bacterial composition of lettuce derived microbiome

| Primer | Sequence 5＇-3＇ |
| --- | --- |
| 338F | ACTCCTACGGGAGGCAGCA |
| 806R | GGACTACHVGGGTWTCTAAT |

**TableS3** Primer sequence for 16S rRNA identification of HBGA-SEB

| Primer | Sequence 5＇-3＇ |
| --- | --- |
| F-primer | CCGGATCCAGAGTTTGATCATGGCTCAGCA |
| R-primer | CGGGATCCTACGGCTACCTTGTTACGACTT |

**TableS4** PCR reaction system of 16S rRNA identification

| 2×PCR Master | 12.5 μL |
| --- | --- |
| F-primer (20 μM) | 1 μL |
| R-primer (20 μM) | 1 μL |
| Template | 1 μL |
| RNase free ddH_2_O | 9.5 μL |
| Total | 25 μL |

After PCR reaction, 16s rRNA gene amplicons were stored at -20 ℃ until sent to Sangon Biotech company(cold-chain transporation) to be sequenced. Amplicon sequences used for HBGA-SEB identification as well as the blasting results were given in Supplementary Materials S4 TableS7. Raw sequences (.fastq) of lettuce leaf surface microbiome was submitted as Supplementary Materials S5_Lettuce leaf surface microbiome.fastq

S3: Quantification of NoVs by RT-PCR

Series of treatments were made based on the viral-bacterial mixture (i.e. by attachment assay) or virus-bacteria-lettuce combination (schematic graph can be seen as FigureS1) to test the effect of simulated environmental conditions on the reduction of NoVs.

The nucleic acid of NoVs was extracted with QIAamp Viral RNA Mini Kit (QIAGEN, CA, Germany) according to the manual book. The information of primers and probes, as well as the RT-PCR reaction procedure are as follows. RT-PCR cycling profile used was 42℃ for 30 min, 95℃ for 2 min, 95℃ for 5 s, 55℃ for 35 s, there are 40 cycles.

**TableS5** Sequence of primers and probes of NoVs

|  | Sequence (5'-3') |
| --- | --- |
| F-primer | CAAGAGTCAATGTTTAGGTGGATGAG |
| R-primer | TCGACGCCATCTTCATTCACA |
| Probe | FAM-AGATTGCGATCGCCCTCCCA-TAMRAR |

**TableS6** RT-PCR reaction system of NoVs

| 2×One Step RNA PCR buffer | 12.5 μL |
| --- | --- |
| Takara Ex Taq HS | 0.5 μL |
| PrimeScript RT Enzyme MiX II | 0.5 μL |
| RNase free ddH_2_O | 5 μL |
| Forward Primer (20 μM) | 0.6 μL |
| Reverse Primer (20 μM) | 0.6 μL |
| Probe (20 μM) | 0.3 μL |
| Template | 5 μL |
| Total | 25 μL |


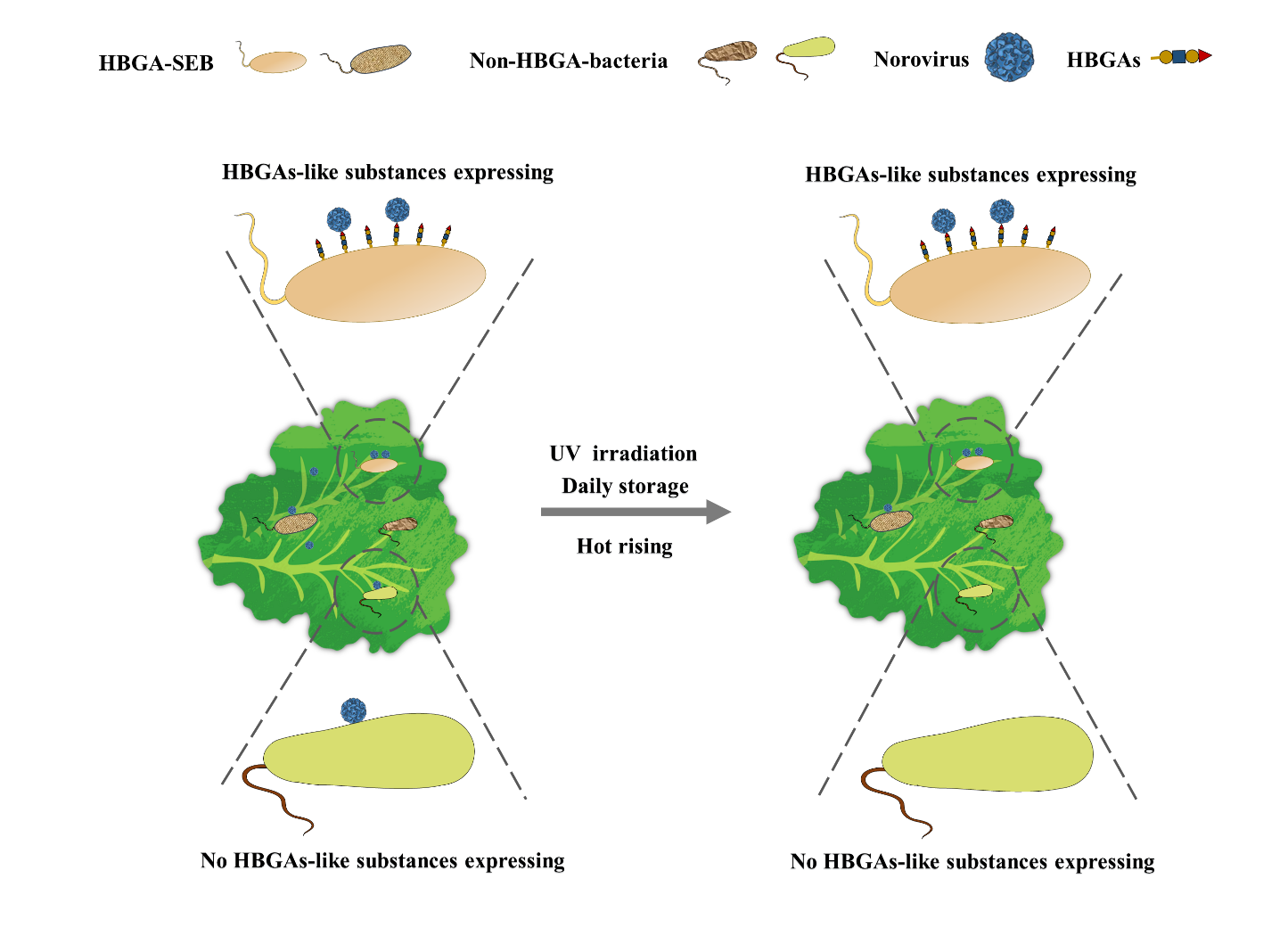


**FigureS1** Schematic graph for testing reduction of NoVs based on virus-bacteria-lettuce combination.
